# Design of solid phase microextraction patches for the detection of volatile metabolites associated with *Burkholderia cepacia*

**DOI:** 10.1007/s00253-026-13962-3

**Published:** 2026-07-15

**Authors:** Sk Rahabar Ali, Debsmita Mandal, Shyam Sundar Nandi, Chiranjit Ghosh, Chiranjay Mukhopadhyay

**Affiliations:** 1 Department of Microbiology, Kasturba Medical College, Manipal Academy of Higher Education, Manipal, India; 2 Manipal Institute of Technology, Manipal Academy of Higher Education, Manipal, India; 3 ICMR-National Institute of Virology, (Mumbai Unit), Indian Council of Medical Research, Haffkine Institute Compound, A. D. Marg, Parel, Mumbai, 400012 India

**Keywords:** *Burkholderia cepacia*, Metabolomics, Lung disease, Sustainable, Thin film solid phase microextraction (TF-SPME), Gas chromatography-mass spectrometry (GC–MS)

## Abstract

**Abstract:**

*Burkholderia cepacia*, a gram-negative bacillus, is widely recognized as an emerging pathogen responsible for nosocomial infections in patients with cystic fibrosis (CF). The conventional culture-based method takes 5–7 days, posing a challenge for rapid bacterial identification. Here, we developed a thin-film solid phase microextraction (TF-SPME) device by coating a fiberglass sheet with hydrophilic-lipophilic balanced (HLB) particles and polydimethylsiloxane (PDMS) polymers, exploiting an automatic film applicator to maintain coating uniformity. The coated sheet was trimmed into multiple microextraction sampling tools, and the individual patches were optimized using a McReynolds standard solution containing benzene, 2-pentanone, pyridine, octane, 1-nitropropane, and 1-pentanol compounds. Headspace sampling was performed to capture the highly volatile compounds, whereas comparatively less volatile and polar compounds were extracted through the direct immersion by the microextraction patches. The TF-SPME patches were desorbed in a gas chromatography triple quadrupole mass spectrometer, and the chromatogram revealed the emission of 34 metabolites associated with *B. cepacia* pathogen during the growth phase. This study demonstrated the feasibility of the utilization of simple, cost-effective microextraction patches for the extraction of *B. cepacia* specific metabolites. Moreover, the greenness assessment score (BAGI score = 70 and AGREE = 0.68) established the sustainability of the sample preparation technique. Further validation with clinical specimen may facilitate the future clinical research for the identification of *B. cepacia*.

**Key points:**

• *Fiberglass-based polymer-coated microextraction patches were fabricated*

• *Fabricated patches were optimized for capturing volatile metabolites*

• *Burkholderia cepacia was screened through in-vitro metabolic profiling by patches*

**Graphical Abstract:**

**Figure:**
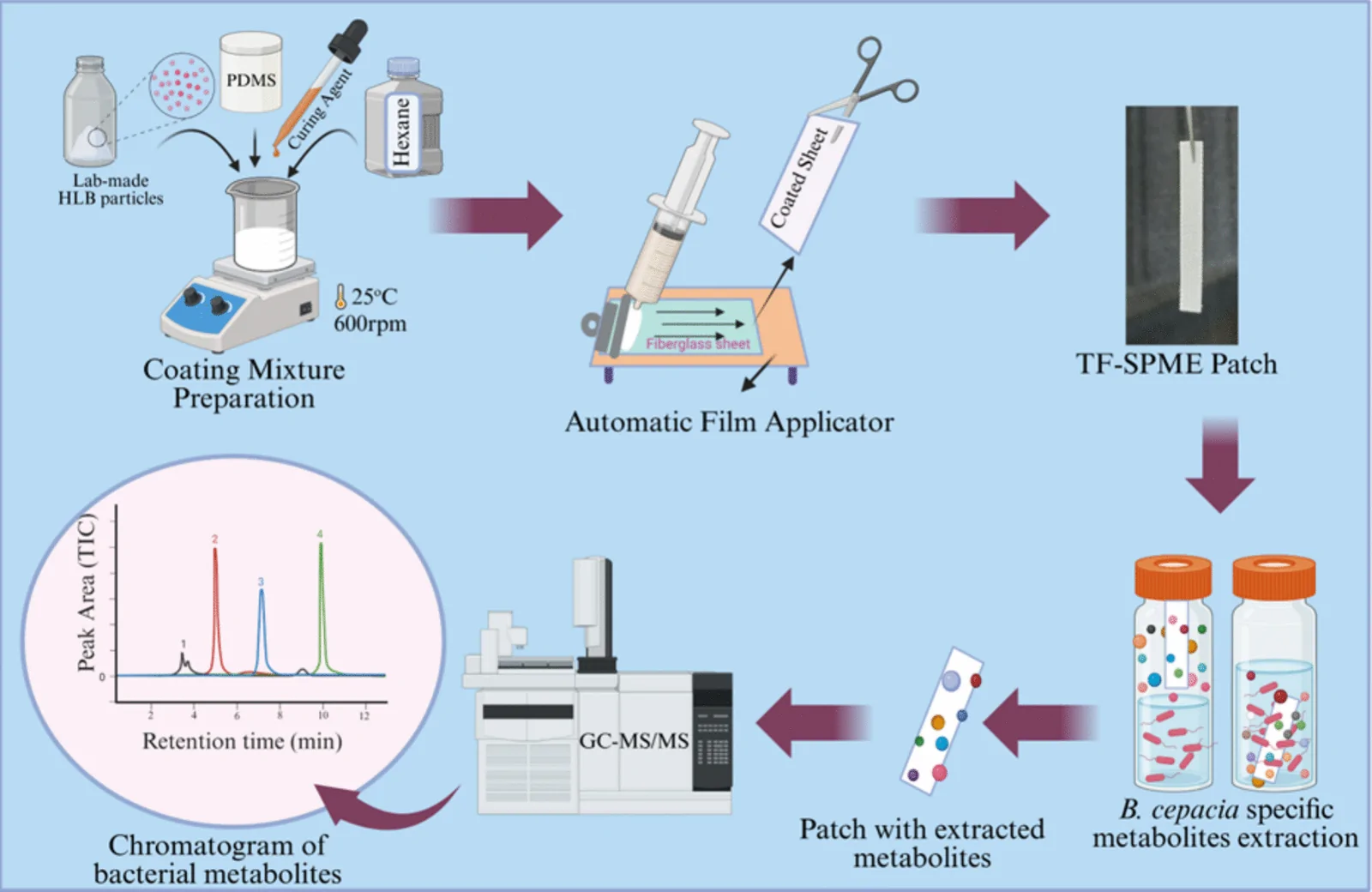
Design of solid phase microextraction patches for the detection of volatile metabolites associated with *Burkholderia cepacia.*

**Supplementary Information:**

The online version contains supplementary material available at https://doi.org/10.1007/s00253-026-13962-3.

## Introduction

*Burkholderia cepacia* is a gram-negative, aerobic, rod-shaped bacterium that is usually found in aquatic environments, fruits, and vegetables, as well as in fluids that are used in hospital settings (Meena et al. 2019; Shaban et al. 2020). It was first discovered in 1947, and later in the 1950 s, it was recognized as a human pathogen that can infect immunocompromised patients with chronic granulomatous disease and cystic fibrosis (CF) (Shmarina et al. 2024). It has been identified as a prevalent pathogen over the past three decades in CF patients (LiPuma 2010). The occurrence of *B. cepacia* infection is not only limited to CF patients but also is associated with nosocomial infections, including ventilator-associated pneumonia (VAP) (Guo et al. 2017), and indwelling device-related infections among Intensive Care Unit (ICU) patients (Liao et al. 2011; Patra et al. 2014). A report on *B. cepacia* bloodstream infections suggested a mortality rate of 24% to 64% among non-CF patients (Shi et al. 2023). Several clinical manifestations have been reported, including sepsis, urinary tract infections, pneumonia, and central nervous system (CNS) infections among children due to *B. cepacia* (Patra et al. 2014). *B. cepacia* is a multidrug-resistant bacterial pathogen; resistance is commonly observed to aminoglycosides, colistin, and gentamicin, which makes infections difficult to treat. Therefore, it is challenging to treat the immunocompromised patients with *B. cepacia* infection, especially in the case of drug resistance of the bacteria (Patra et al. 2014).

The conventional culture methods are usually used to identify *B. cepacia*, but it takes from 5 to 7 days for the isolation and identification (Tavares et al. 2020; Ahn et al. 2020). Some commercial methods, including API 20NE, Vitek GN1/GN1 PLUS, MicroScan analysis, and RapID NF plus, are used to identify *B. cepacia* complex (BCC) from the cultures. These techniques are expensive and sometimes responsible for false negative results, along with low sensitivity (Ahn et al. 2020). Contemporary molecular techniques, such as quantitative real-time polymerase chain reaction (qPCR) and *recA*-based PCR, were employed to isolate *B. cepacia* directly from clinical samples (Liu et al. 2024). However, the practical applications of these techniques are limited due to high cost and lack of sensitivity (Daddy Gaoh et al. 2023). Furthermore, the PCR-based techniques provide information about the bacterial species arising from both live and dead cells (Priyanka et al. 2016), which may enhance the chances of false positives and inaccurate quantification of the active bacterial load. Therefore, there is a precise need for the development of a rapid, cost-effective, and reliable pathological technique for the identification of *B. cepacia.*

To address these issues, various analytical techniques were proposed by investigators for the determination of bacterial pathogens from the culture study. Solid phase microextraction (SPME) devices emerged as a potential and sustainable sample preparation technique for monitoring volatile emissions from the bacterial culture media (Selvamuthukumar et al. 2025). The application of SPME has been extended to determine the volatile and semi-volatile compounds for environmental research, biomedical applications, pharmaceutical analysis, *in vitro* and *in vivo* metabolomic studies (Reyes-Garcés et al. 2018). However, the SPME fiber is fragile in nature and prone to physical damage during handling (Magalhães et al. 2026). Moreover, the limited surface area of the fiber saturates quickly while analyzing a complex matrix or a multicomponent mixture. This limits the accurate quantification of a compound in a multicomponent mixture, because analytes compete for the limited binding sites. In addition, the SPME fiber can exhibit biases towards certain compounds depending on the chemical nature and polarity of the coating materials, such that analytes with stronger interaction with the sorbent outcompete those with weaker interactions (Kabir and Kim 2012; Illanad et al. 2024). The lower recovery of the compound and biases towards certain compounds make it less effective for clinical sample preparation. To overcome these issues, scientists developed thin-film solid phase microextraction (TF-SPME), another geometry of SPME with a high surface area-to-volume ratio and flexibility for potential extraction of volatile compounds from various matrices (Murtada and Pawliszyn 2022). These TF-SPME tools are advantageous to extract the analytes both from the headspace (HS) and direct immersion (DI) modes (Jalili et al. 2020). In addition, this microextraction technique requires minimal solvent and integrates both the sampling and preconcentration in a single step that eliminates the need of separate extraction step, which typically requires a large volume of organic solvent. So, minimizing solvent consumption and reducing sample preparation steps lowers the environmental impact, aligning with the principle of green analytical chemistry. This green sample preparation technique ensures environmental sustainability in medical research (Olcer et al. 2019; Murtada and Pawliszyn 2022).

The TF-SPME patches work on the principle of mass transfer of analytes from the sample matrix to the coating substrate until equilibrium is reached (Jiang and Pawliszyn 2012). Filipiak et al. 2024 used hydrophilic-lipophilic balance (HLB) particles coated TF-SPME patches to extract volatile organic compounds (VOCs) from the bronchoalveolar lavage (BAL) samples of VAP patients (Filipiak et al. 2024). However, the application of TF-SPME in a diagnostic laboratory is minimal because of the high cost of the commercial patches. Therefore, there is a research gap to develop cost-effective TF-SPME tools for clinical research.

To address the high cost of the commercial TF-SPME and identification of *B. cepacia* pathogen-specific metabolites, fiberglass-supported HLB/PDMS-based TF-SPME patches were developed using in-house coating materials. The fabricated patches were optimized with McReynolds standard solutions. After that, the microextraction devices were exposed to the *B. cepacia* culture media using direct immersion and headspace to extract polar and non-polar compounds emitted from the culture media. Our study showed that a fiberglass-based simple analytical microextraction device can be an alternative way for the screening of *B. cepacia* through monitoring of volatile emissions of the bacteria. This may serve as a potential technique for application in a diagnostic laboratory where PCR technology is not easily available, or it is required for the rapid identification of the active *B. cepacia* bacterial species in biological samples.

## Materials and methods

### Chemicals and materials

Divinylbenzene (monomer), N-vinylpyrrolidone, high-density polydimethylsiloxane (PDMS), benzene, and 1-nitropropane were bought from Sigma-Aldrich (Bangalore, India). LOBA Chemie Pvt Ltd (Colaba, Mumbai, India) supplied the acetonitrile (ACN), ethyl acetoacetate, 2-pentanone, isopropyl alcohol, 2-azobis(isobutyronitrile), and 1-pentanol. Hexane, octane, and pyridine were purchased from MERCK, Spectrochem, and Molychem, respectively. Ethanol was supplied by Jiangsu GTIG Huatai Co., Ltd (Taicang port development zone, China). Methanol was bought from Finar (Ahmedabad, Gujarat, India), and plain glass fabric (200 GSM fiberglass) was bought from The Bohr Chemicals and Plastics Pvt Ltd (Nashik, Mumbai, India). Nitrogen gas (N_2_) was obtained from the West Coast Gas Agency (Udupi, Karnataka, India). Ultrapure water was obtained from the Merck Milli-Q purification system. HiMedia (Thane, Maharashtra, India) supplied the 5% sheep blood agar plate and tryptone soya broth. The 40-mL glass vials were obtained from Sigma-Aldrich (Bangalore, India), and TF-SPME fiber holding clips were bought from the local market.

### Fabrication of HLB-coated TF-SPME patches

**Fig. 1:**
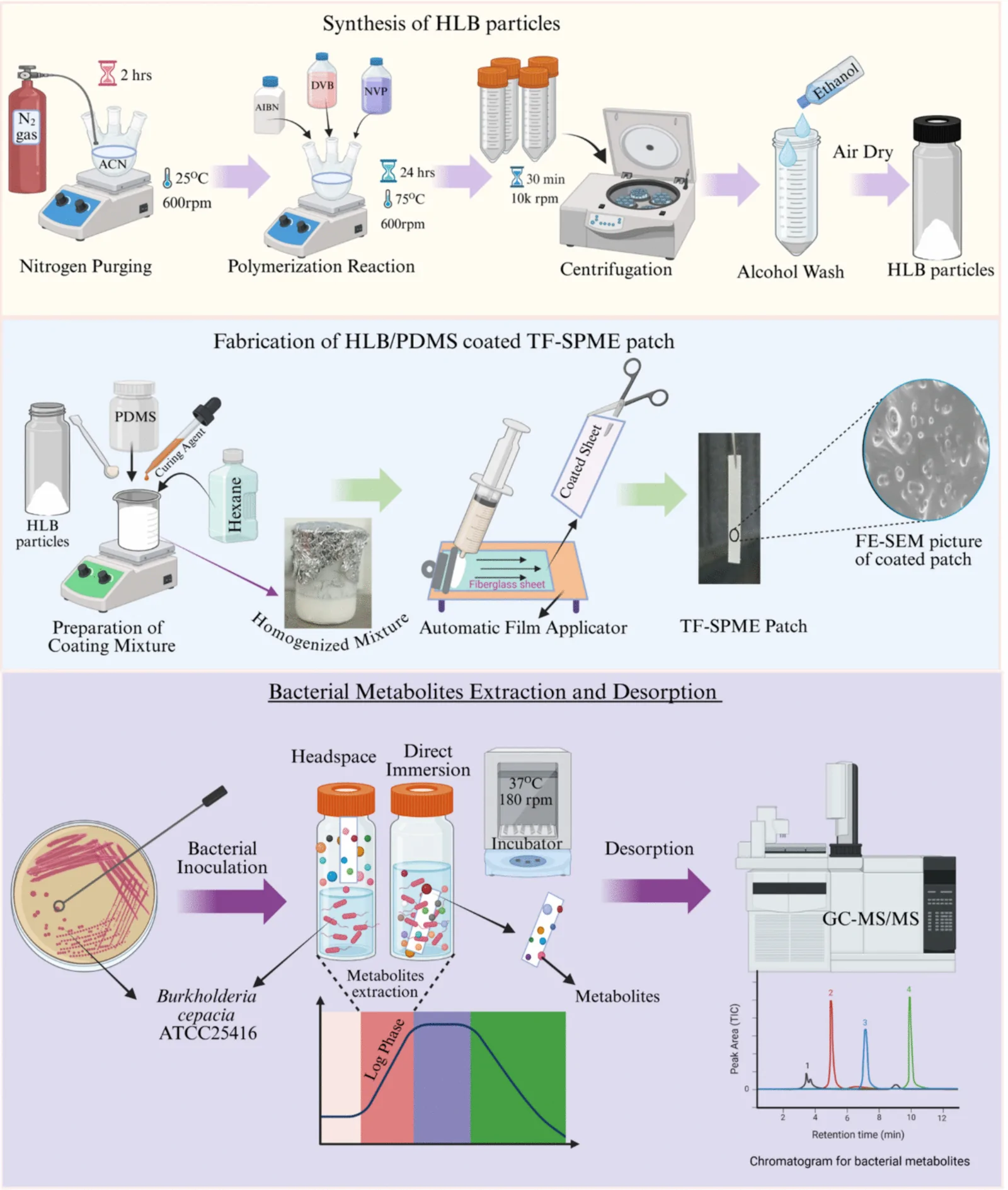
Pictorial overview of the experiment. **a** Synthesis of hydrophilic lipophilic balance (HLB) particles. **b** Fabrication of HLB/PDMS-coated TF-SPME patch. **c** Bacterial metabolites extraction and desorption. (ACN acetonitrile, AIBN 2-azobisisobutyronitrile, DVB divinylbenzene, NVP N-vinylpyrrolidone, PDMS polydimethylsiloxane)

Development of the TF-SPME patches comprises three steps, including the synthesis of HLB particles, coating of the HLB/PDMS mixture on plain glass fabric, and trimming of the coated sheet into the desired shape. In the first step (Fig. 1a), ACN was purged with N_2_ gas for 2 h to minimize the presence of oxygen. To start the polymerization process, n-vinylpyrrolidone (NVP) monomer and divinylbenzene (DVB) crosslinker were mixed with N_2_ purged ACN and then 2-azobisisobutyronitrile (AIBN) was added as initiator into the resultant solution. Then the mixture was kept overnight at 75 °C with a 600 rpm stirring rate to initiate the polymerization reaction. The solution was centrifuged at 10,000 rpm for 30 min at 4 °C to get the HLB polymer particles. After that, ethanol was used to wash the polymer particles. In the next step (Fig. 1b), synthesized HLB particles were mixed with 25 to 35 vol/vol % concentrated PDMS, curing agent, and hexane to make a homogenized mixture. PDMS is known to have chemical stability and flexibility with HLB particles (van der Merwe and Forbes 2024). The homogenized mixture was coated on both sides of the plain glass fabric using an automatic film applicator (Elcometer 4340), where we kept a 70 µm coating thickness. The vacuum oven was used to dry the coated sheet under a N_2_ environment to remove the contaminants. The coated sheet was cut into 0.5 × 4 cm pieces to obtain multiple disposable microextraction patches.

### Instrumentation

An Agilent 8890 gas chromatography and 7000E MS/MS detector was used for the separation and detection of the compounds. In this study, an Agilent DB624 column with a length of 30 m, an internal diameter of 0.25 mm, and a film thickness of 1.40 µm was used for chromatographic separation. The temperature of the injector port was maintained at 220 °C and the transfer line temperature at 240 °C with a m/z scan range of 40 to 500. As a carrier gas, helium was used at a constant flow rate of 1.2 mL/minute. Agilent PAL-3, a robotic automation tool, was used to inject the samples into the injector port of the GC–MS/MS system. The Agilent PAL-3 injected 1 µL of the samples into the injector port of the GC–MS/MS system. The GC oven temperature was initially maintained at 40 °C for 4 min and ramped at a rate of 10 °C/min up to 240 °C, where it was held for 10 min.

An automatic film applicator, Elcometer 4340 (Manchester, UK), was used for uniform coating on the fiberglass surface. The magnetic stirrer (Model: 2MLH) and shaking incubator (CIS-18) were procured from REMI (Mumbai, Maharashtra, India). For microbiological works, a Biological Safety Cabinet Class A2 (Model: 1584, Thermo Scientific, Suzhou, Jiangsu, China) was utilized for the study.

### Validation of developed TF-SPME patches

#### Extraction of McReynolds standard by fiberglass patches

To optimize the fabricated HLB/PDMS patches, we used the McReynolds standard containing 2-pentanone, benzene, pyridine, 1-nitropropane, octane, and 1-pentanol analytes. The compounds were spiked into hexane to obtain the main stock at 1000 ng/mL. The disposable patches were then exposed to the 20 mL hexane-based McReynolds standard solution, and extraction was performed at room temperature (25 °C) for 30 min with a rotation speed of 180 rpm. The analytes were extracted at the same temperature (25 °C) in two different modes (direct immersion and headspace) with two separate patches, and all patches were discarded after a single use. After the extraction, the patch was dipped into 2 mL of ACN in a glass vial, and it was kept in an incubator at 45 °C for 30 min with a shaking speed of 200 rpm, in order to desorb the analytes from the patch. The GC–MS/MS equipment was utilized to quantify the analytes after extraction from the standard solution.

#### Optimization of temperature parameter during extraction by HLB/PDMS patches

To investigate how the temperature influences the extraction efficiency of the developed patches, we directly immersed the patch in the McReynolds standard at different temperatures (5 °C, 15 °C, 25 °C, 35 °C, and 45 °C) for 30 min. To perform the experiment, we prepared a 1000 ng/mL McReynolds standard, and the fabricated patches were directly immersed in the standard solution at various temperatures for 30 min at 180 rpm. After the extraction, the patch was dipped into 2 mL of ACN in a glass vial, and it was kept in an incubator at 45 °C for 30 min with a shaking speed of 200 rpm, in order to desorb the analytes from the patch. In this experiment, the mentioned desorption temperature was used for desorption of analytes from the patch.

Similarly, after extracting McReynolds standard (1000 ng/mL) using developed HLB/PDMS patches at 25 °C for 30 min, the analytes were desorbed into ACN at different temperatures (25 °C, 30 °C, 35 °C, 40 °C, and 45 °C) for 30 min to investigate the influence of temperature on the desorption. The analytes were relatively quantified by the multiple reaction monitoring (MRM) mode of GC–MS/MS.

#### Characterization of sorbent particles and developed TF-SPME patches

The size and shape of the synthesized HLB particles and the morphology of the developed HLB/PDMS patches were determined with a CARL ZEISS (USA) field emission scanning electron microscope (FE-SEM). The FE-SEM images were obtained at a 6.4 mm working distance with a 5 kV accelerating voltage and 10 KX magnification. The energy dispersive X-ray spectroscopy (EDX)-Nano XFlash detector, Bruker (Germany) was used to investigate the presence of elemental composition in HLB particles. The EDX image was obtained at a working distance of 9.8 mm with a 2500 × hν magnification and an image resolution of 20 µm. The infrared spectroscopic data of HLB particles were obtained from a Fourier transform infrared spectroscopy (FT-IR) spectrometer (Bruker Tensor 27).

#### Mechanical stability and thermal stability of the patch

Hitachi STA7200 was used to investigate the thermal stability of the developed patches. The patch was kept on an alumina crucible and heated at a temperature range of 30 to 900 °C with a heating rate of 10 °C/min. The mass loss was continuously recorded to assess the thermal stability of the patch.

A universal testing machine (UTM) by Shimadzu-EZ-SX with a suitable tensile gripper was used to check the mechanical stability of the patch. The patch was placed between the upper and lower grips with vertical alignment, while the distance between the grips (gauge length) was 50 mm. The tensile strength of the specimen was tested at room temperature with a continuous crosshead speed of 10 mm/min. The constant record of applied force (N) and stroke (mm) was obtained using the data acquisition software.

### Bacterial culture and extraction of metabolites

The *Burkholderia cepacia* (ATCC 25416) bacterial strain was grown in 5% sheep blood agar at 37 °C overnight. After culturing, the bacterial species was confirmed by MALDI-TOF MS (bioMérieux). Then, the bacteria were subcultured into 5% sheep blood agar. Bacteria from the subculture were inoculated into 160 mL of tryptone soya broth (TSB) to achieve an optical density (OD_600_) of 0.1. Then the bacteria-inoculated broth was aliquoted into eight different 40 mL vials. Four individual replicates were used for headspace analysis and direct immersion experiments. Two 40 mL vials containing 20 mL TSB broth were used as controls (without any bacteria) for both direct immersion and headspace. The developed (HLB/PDMS) patches were placed in the headspace region and within the media to collect preconcentrated volatile compounds. The patches were exposed to the culture medium at 37 °C with constant stirring (180 rpm) until the optical density (OD_600_) of the *B. cepacia* culture reached 0.1–0.5, which took 8 h. After the extraction, the patches were dipped into 2 mL of ACN in a glass vial, and those were kept in a shaking incubator at 45 °C for 30 min with a shaking speed of 200 rpm, in order to desorb the metabolites from the patches to the ACN solvent. The desorption volume of 2 mL was selected based on complete immersion of SPME patches and to ensure efficient analyte desorption. The optimal extraction (37 °C) and desorption temperature (45 °C) were the same for both direct immersion and headspace. The desorbed metabolites were analyzed through GC–MS/MS scan mode. Agilent MassHunter Qualitative software (B.07.00) was used to extract and identify raw GC–MS/MS data. Figure 1c shows the schematic representation of the experiment. Before bacterial metabolite extraction, the patches were optimized by using the McReynolds standard to evaluate their extraction performance.

### Environmental sustainability of this technique

To assess the greenness of this technique, the Blue Applicability Grade Index (BAGI) score was measured by using https://bagi-index.anvil.app/ software based on some parameters listed in Table S1. As well as the AGREE-Analytical GREEnness assessment calculator was used to assess the greenness of this technique (parameter listed in Figure S1). The AGREE software is freely available for download at https://mostwiedzy.pl/AGREE.

### Statistical analysis

The Origin Pro software was used for the statistical analysis, and the resulting plots were included in the manuscript. The peak areas obtained from GC–MS/MS were directly used for statistical analysis without normalization. The data were presented as mean ± standard error. For patch validation, the study was performed with five replicates for all parameters. In this study, the peak area was used as a performance indicator of extraction efficiency as all the studies were performed under identical analytical conditions. Also, blank samples were run to understand the absolute response from the sample matrix. The compounds reported from the bacteria-inoculated culture media were compared with those from the control culture media, which were not inoculated with bacteria. OriginPro software was used to plot the figures and calculate the statistical parameters.

## Results

### FE-SEM and EDX analysis

**Fig. 2:**
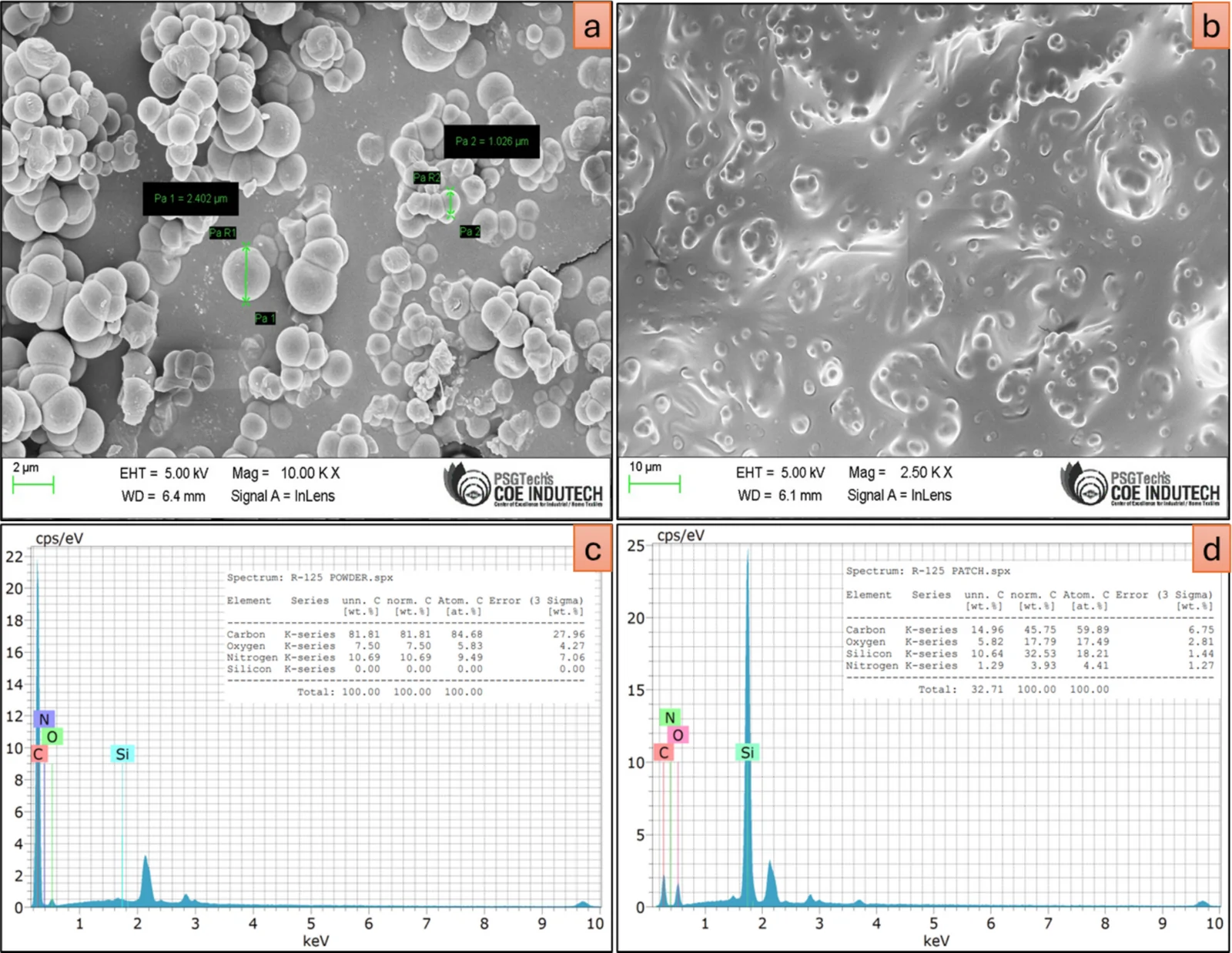
FESEM and EDX results. **a** FESEM image of synthesized HLB particles. **b** FESEM image of HLB/PDMS-coated developed patch. **c** EDX analysis of HLB particles. **d** EDX analysis of HLB/PDMS-coated developed patch

The FE-SEM image showed that the synthesized HLB particles were within the range of 1–5 µm in diameter (Fig. 2a) and were uniformly coated on the patch (Fig. 2b). The elemental composition data from EDX analysis clearly showed the presence of carbon (C), nitrogen (N), and oxygen (O) at 84.68%, 9.49%, and 5.83%, respectively, in HLB particles (Fig. 2c). The EDX data indicated that the particles’ major components were carbon atoms. The nitrogen signal in the EDX data was attributed to the N-vinylpyrrolidone group in HLB particles, and the minor oxygen enrichment was due to surface oxidation and impurities. The EDX data of the HLB/PDMS patch showed the presence of silicon (Si) at 18.21% along with carbon (59.89%), nitrogen (4.41%), and oxygen (17.49%) (Fig. 2d). The presence of HLB particles makes a porous network that promotes the adsorption of analytes.

## FT-IR analysis

**Fig. 3:**
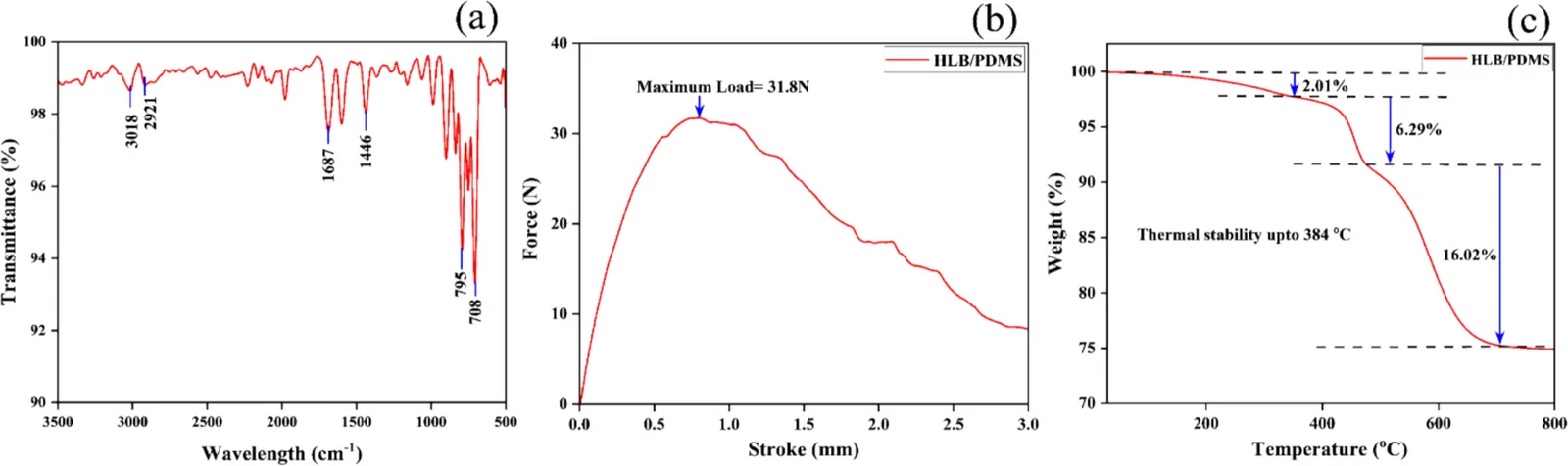
**a** FT-IR spectrum of synthesized particles. **b** Universal testing machine (UTM) analysis of the developed HLB/PDMS patch. **c** Thermogravimetric analysis of the developed HLB/PDMS patch

FT-IR analysis of HLB particles was done to identify the unknown compounds from the spectra, which are specific to the functional groups. In Fig. 3a, the spectrum peak range of 2848–2921 cm^−1^ was attributed to C–H stretching of methylene, whereas 3084–3018, 795–708, and 1642–1446 cm^−1^ were attributed to aromatic C = C–H, aromatic C–H bending, and C–C stretching, respectively. Moreover, the 1687 cm^−1^ peak was assigned to the C = O stretching vibration of N-vinyl pyrrolidone. These prototype peaks of absorption clearly define the formation of poly resin (divinylbenzene-co–N-vinylpyrrolidone).

## Mechanical stability testing

To check the mechanical stability of the developed TF-SPME patches, we performed tensile testing using a UTM by Shimadzu-EZ-SX. The UTM data (Fig. 3b) demonstrates that the fiberglass-based developed patch endures a maximum load of 31.8 N force. This result depicts that the developed patch is mechanically stable and feasible to use in the analytical sample preparation process on a regular basis.

## Thermogravimetric analysis

The thermogravimetric analysis (TGA) was investigated to assess the thermal stability of the developed patches. The patch (13.89 mg) was checked at a temperature range of 30–900 °C with a 10 °C ramp temperature. The data indicates that the patch started to degrade at 384 °C and continued till the second degradation with 6.29% weight loss. After the commencement of the second degradation, 16.02% of weight loss occurred between 482 and 700 °C, which led to the complete decomposition of the polymer. Figure 3c shows that the developed patch remained stable at temperatures up to 384 °C.

## Relative extraction performance of the developed patches

The HLB/PDMS-based microextraction device was designed for the balanced extraction of polar and non-polar analytes. The McReynolds standard (mentioned in Table 1) provides an appropriate matrix with a wide range of polar to non-polar analytes to optimize the relative extraction performance of the patch (Grandy et al. 2018).

**Table 1:** McReynolds standard

Sl. No.	McReynolds standard	Log [*P*] value	Bolling point (°C)
1	Benzene	2.13	80.1
2	2-pentanone	0.98	102
3	Pyridine	0.84	115.2
4	Octane	4.78	125.6
5	1-nitropropane	0.94	132.0
6	1-pentanol	1.7	138.0

**Fig. 4:**
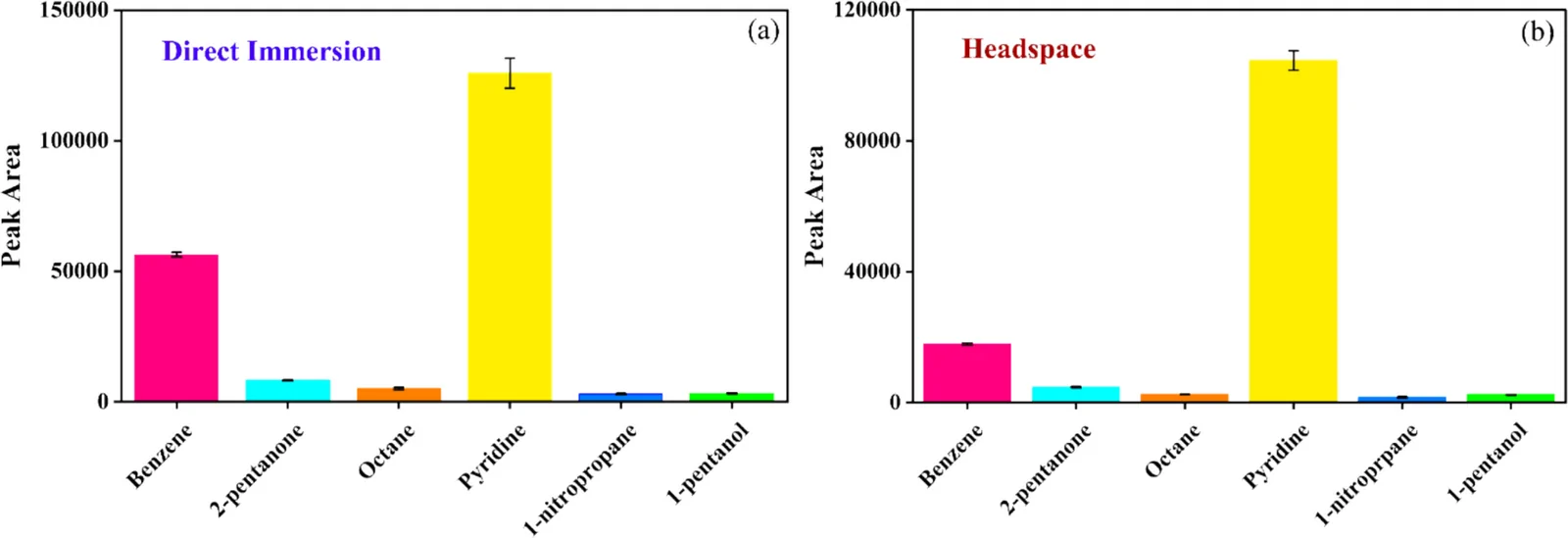
Validation of the developed patch. **a** Relative extraction efficiency of the developed patch in direct immersion. **b** Relative extraction efficiency of the developed patch in the headspace. (Error bar represents the standard error of the mean)

Extraction of McReynolds standard (1000 ng/mL) was performed at 25 °C with a 180 rpm stirring rate, using the developed patch (HLB/PDMS) to assess extraction efficiency. The result (Fig. 4) demonstrated that the developed patch is compatible with extracting the compounds of McReynolds standard in the hexane matrix. Among the compounds, pyridine was extracted in the highest amount, followed by benzene, 2-pentanone, octane, 1-pentanol, and 1-nitropropane. The compounds were extracted in a similar fashion for both direct immersion and headspace (Fig. 4a, b). In direct immersion, compounds were extracted more readily because of the direct contact of the sorbent coating with the sample matrix, which enhances the mass transfer of the analytes from the sample to the extraction phase. As well as the direct contact increases the partitioning of compounds into the sorbent and minimizes the diffusion barrier, that leads to better recovery of the compounds.

## Influence of temperature on the extraction and desorption efficiency of developed patches

**Fig. 5:**
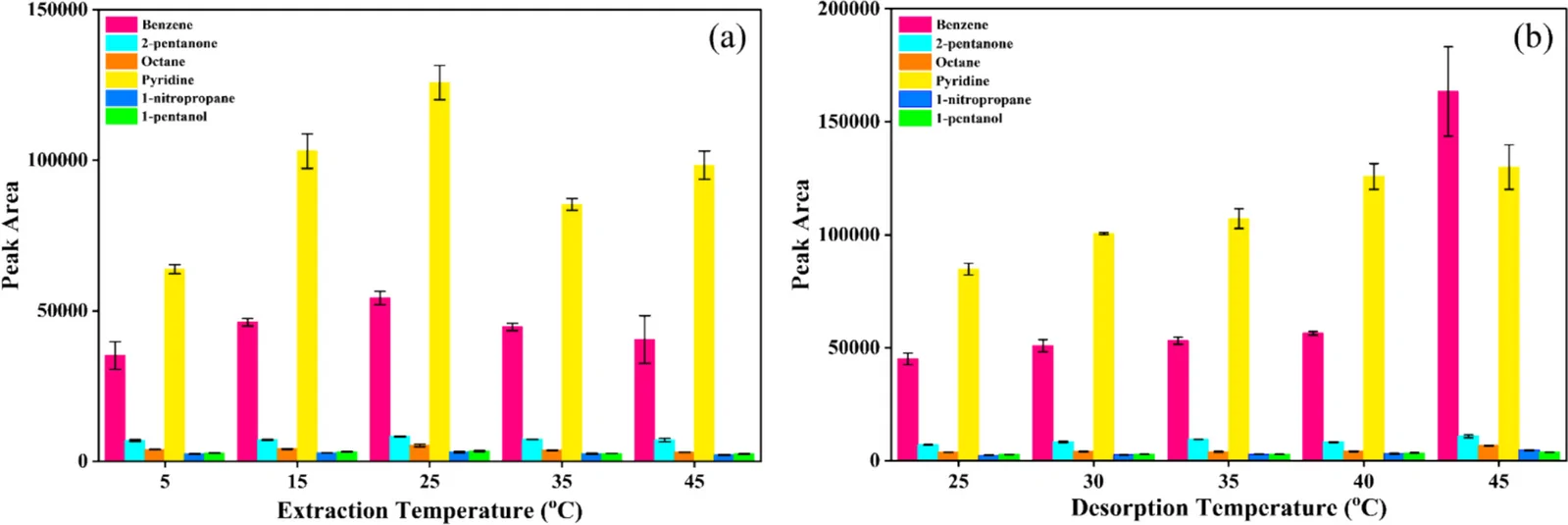
Temperature profile of developed patches. **a** Influence of temperature during extraction. **b** Influence of temperature during desorption. (Error bar represents the standard error of the mean)

To investigate how temperature influences the extraction of the developed patches, McReynolds standard (1000 ng/mL) was extracted at 5 °C, 15 °C, 25 °C, 35 °C, and 45 °C, with desorption at 45 °C, to assess the impact of temperature. Moreover, the same McReynolds standard was extracted at 25 °C and desorbed at different temperatures (25 °C, 30 °C, 35 °C, 40 °C, and 45 °C) to observe how temperature affects the desorption. Figure 5a shows that the extraction efficiency increased with increasing temperature up to 25 °C. The patch extracted the highest at 25 °C. Beyond that temperature, the extraction efficiency starts to decrease. Increasing the temperature increases the diffusion coefficient of analytes in the sample matrix, accelerating their mass transfer to the TF-SPME membrane and decreasing the equilibrium time. However, at higher temperatures, the extraction of the analytes decreases because of the exothermic nature of the sorbent (HLB) particles. High thermal energy weakens the intermolecular forces between the sorbent and the analytes in the sample matrix, thereby shifting the equilibrium towards desorption. On the other hand, the desorption rate increases with temperature, reaching a maximum at 45 °C, while it is lower at lower temperatures (Fig. 5b).

## Assessment of environmental sustainability of the proposed technique 

**Fig. 6:**
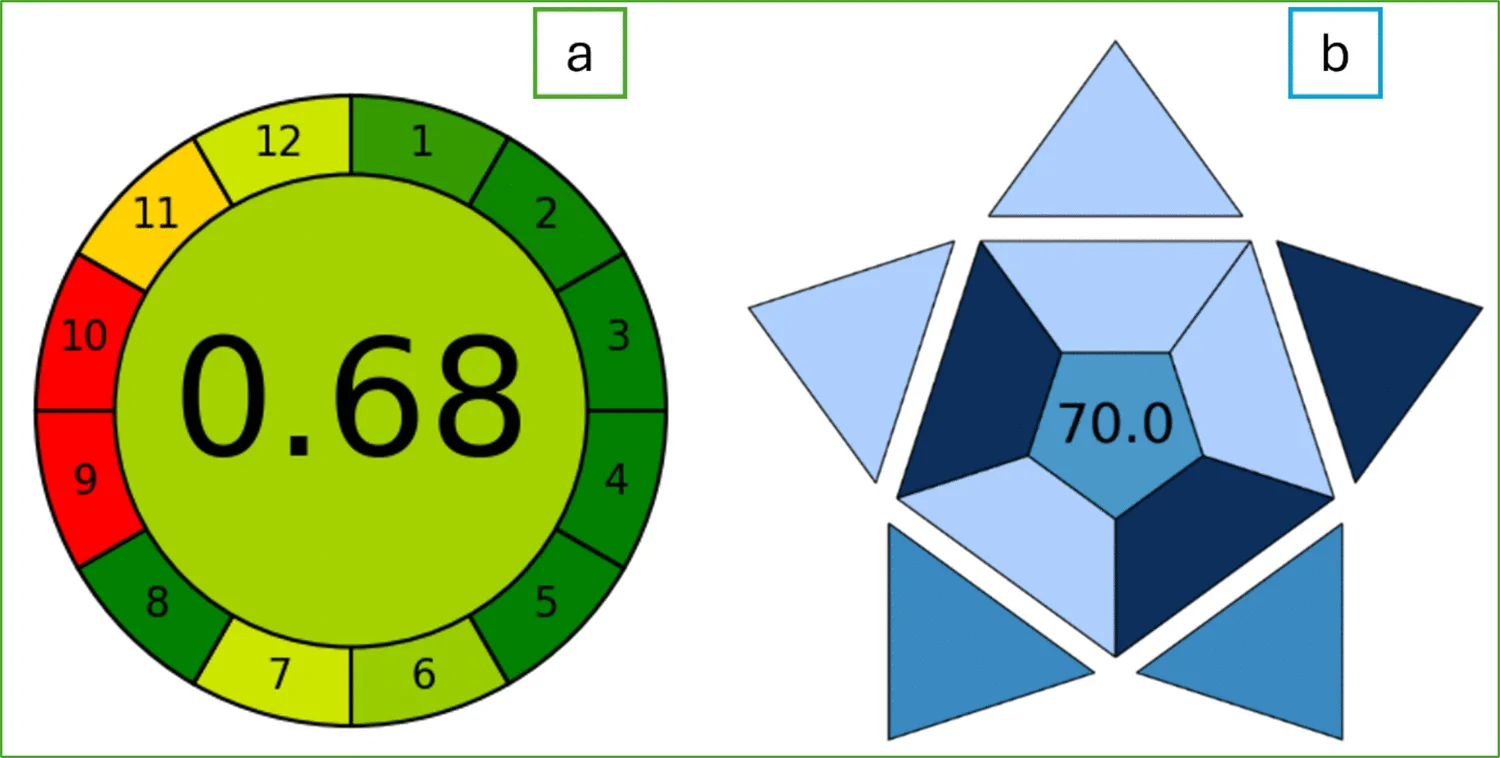
Greenness assessment score of the technique. **a** AGREE-analytical GREEnness assessment score. **b** Blue applicability grade index (BAGI) score

The blue applicability grade index (BAGI) score was calculated depending on several factors (Table S1) to determine the environmental sustainability of this technique. The recommended BAGI score is more than 60 to make any analytical technique useful for green assessment (Manousi et al. 2023). The approximate BAGI score for this proposed technique was 70.0, indicating its eco-friendliness. In the pictogram (Fig. 6b), deep blue, medium blue, light blue, and white demonstrate high, medium, low, and no compliance, respectively. On the other hand, the AGREE-Analytical GREEnness assessment score is 0.68 (Fig. 6a), with deep green indicating the highest and red the lowest. The AGREEprep (Analytical Greenness Metric for Sample Preparation) report is also attached in the supplementary materials (Figure S1).

## Bacterial culture emitted metabolites

The developed patches were used to extract metabolic emissions from *B. cepacia* bacterial culture media. The data expressed in Table 2 represent the *B. cepacia*-emitted metabolic profile after exclusion of the broth-emitted compounds. The emission of volatile and semi-volatile compounds was collected during the log phase of the bacterial growth through headspace and direct immersion. The GC–MS/MS detected a total of 29 compounds. Each compound was present in more than two replicates of the experiment and absent in the control medium to eliminate media-emitted compounds. Among these putatively identified compounds, eleven compounds were from headspace, and eighteen were from direct immersion, whereas four metabolites were common in both direct immersion and headspace, as demonstrated in Figure S2b. These four metabolites are 1,3-dimethylbenzene, tetramethylbutanedinitrile, vinyl cinnamate, and 2,4-dimethylphenyl acetaldehyde.

**Table 2:** Metabolites emitted from *Burkholderia cepacia* culture medium

Compounds name	Formula	Molecular weight (g/mol)	Base peak (m/z)	NIST match factor	RT (min)	Category
Compounds from headspace						
1,3-dimethylbenzene	C_8_H_10_	106.16	91	89.8	11.87	Heteroaromatics
2,6-dimethyl-3-pyridinamine	C_7_H_10_N_2_	122.16	122	71.7	13.71	Others
4,5-dimethylnonane	C_11_H_24_	156.31	71	75.9	13.9	Others
1-ethenyl-4-ethylbenzene	C_10_H_12_	132.20	117	98.1	14.73	Heteroaromatics
Tetramethylbutanedinitrile	C_8_H_12_N_2_	136.19	69	89.7	14.89	Others
Vinyl cinnamate	C_11_H_10_O_2_	174.2	131	87.5	16.28	Acid
3-Ethyl-3-methylheptane	C_10_H_22_	142.28	57	70.6	16.33	Others
2,4-dimethylphenyl acetaldehyde	C_10_H_12_O	148.2	119	74.3	16.69	Aldehyde
3,3,5-trimethyldecane	C_13_H_28_	184.36	71	73.7	16.77	Others
2-ethylhexyl hexyl sulfite	C_14_H_30_O_3_S	278.45	57	73	17.9	Others
7-methylpentadecane	C_16_H_34_	226.44	57	78.2	19.55	Others
Compounds from direct immersion						
Acetoin	C_4_H_8_O_2_	88.11	45	93.5	10.08	Ketone
1,3-dimethylbenzene	C_8_H_10_	106.16	91	94.4	11.87	Heteroaromatic
2,6-dimethylpyrazine	C_6_H_8_N_2_	108.14	108	78.8	12.57	Heteroaromatic
4,6-dimethylundecane	C_13_H_28_	184.36	57	74.1	13.9	Others
2,6-diethylpyrazine	C_8_H_12_N_2_	136.19	135	90.1	14.58	Heteroaromatic
3-ethyl-2,5-dimethylpyrazine	C_8_H_12_N_2_	136.19	135	85.1	14.58	Heteroaromatic
Tetramethylbutanedinitrile	C_8_H_12_N_2_	136.19	69	91.1	14.9	Others
Acetophenone	C_8_H_8_O	120.15	105	72.9	14.97	Ketone
Vinyl cinnamate	C_11_H_10_O_2_	174.2	131	88.4	16.27	Acid
3,4-dimethylbenzaldehyde	C_9_H_10_O	134.17	133	84.6	16.71	Aldehyde
1-methyl-4-(1-methylpropyl)-benzene	C_11_H_16_	148.24	119	77.5	16.92	Heteroaromatic
2,4-dimethylphenyl acetaldehyde	C_10_H_12_O	148.20	119	72.4	16.92	Aldehyde
1-(4-ethylphenyl) ethanone	C_10_H_12_O	148.20	133	73.4	17.06	Ketone
1-ethyl-2,3-dihydro-1H-Indene	C_11_H_14_	146.22	117	74	17.17	Others
Benzene acetaldehyde, alpha-ethylidene-	C_10_H_10_O	146.19	117	90.8	17.31	Aldehyde
1-iodododecane	C_12_H_25_I	296.23	57	78.6	17.42	Others
2,6,11-trimethyldodecane	C_15_H_32_	212.41	57	82.5	18.42	Others
2,4-dihydroxy-6-methylbenzaldehyde	C_8_H_8_O_3_	152.15	151	81.3	19.09	Aldehyde

In this research work, the metabolites were putatively identified after comparison of the experimental data with the NIST spectral library. The compounds were mainly selected based on manual interpretation of the base peaks and fragment ions and later matched against the NIST database. The compounds listed in Table 2 were classified into different categories, including ketones, acids, heteroaromatics, alcohols, aldehydes, and other compounds, including the aliphatic hydrocarbons (alkane, alkene, alkyne). Figure S2a demonstrates the detailed classification of the compounds. The presence of acetoin in the volatile emissions from the culture media was found to match the *B. cepacia-*specific metabolic profile reported by previous works (Dryahina et al. 2016). Being a polar molecule, acetoin (3-hydroxy-2-butanone) could easily bind to the hydrophilic region of HLB particles. Apart from acetoin, several other volatile and semi-volatile compounds, including acetophenone (Gutiérrez-Santa Ana et al. 2020), 3-ethyl-2,5-dimethylpyrazine, 2,6-diethylpyrazine (Fitzgerald et al. 2020; Dixon et al. 2022), pyridine, 1-iododecane, 2,6-dimethylpyrazine, ethylbenzene, and 1,3-dimethylbenzene (Timm et al. 2018; Davis et al. 2020) were reported as microorganism-specific metabolites by several studies.

## Discussion

The study aimed to develop and thereafter validate a cost-effective, eco-friendly microextraction patch for capturing the bacteria-associated volatile and semi-volatile compounds during the bacterial growth phase. Here, *B. cepacia* was used as a model organism as it is challenging to identify this bacterium in clinical settings. The TF-SPME patch was developed by utilizing laboratory-synthesized HLB particles for balanced extraction of both polar and non-polar metabolites from the bacterial culture. The microporous HLB particles were synthesized by precipitation polymerization and coated on the surface of fiberglass in combination with PDMS. The FE-SEM images demonstrated the integration of microporous HLB particles into the thin film support, and the coating was consistent and continuous. The spherical morphology of the particles and several interconnected pores are the characteristics of the divinylbenzene-co-n-vinylpyrrolidone (HLB copolymer). The microporous and mesoporous network significantly contributed to analyte adsorption, thereby increasing the extraction capacity. In comparison with the traditional SPME fiber, this developed TF-SPME patch had a high surface area (Krumplewski and Rykowska 2024). The HLB copolymer (divinylbenzene-co-n-vinylpyrrolidone) was synthesized by the precipitation polymerization method, producing a porous polymer network with several interconnected pores (Nie et al. 2022). However, the Brunauer–Emmett–Teller (BET) surface area analysis was not performed in this study. In future work, surface area measurement will be required for quantitative measurement of the surface area of HLB particles. On the other hand, the elemental composition has been measured by EDX analysis, where the nitrogen (N) peak supports the inclusion of NVP, which increases hydrophilicity and allows dipole–dipole interactions and hydrogen bonding. The richness in carbon (C) and nitrogen/oxygen contributes to both hydrophilic and hydrophobic interactions. The detection of silicon (Si) atoms in patches indicated the presence of PDMS as a supporting material.

The distinctive absorption bands from the FT-IR analysis of the synthesized particles corresponded to the symbolic functional groups of the divinylbenzene-N-vinylpyrrolidone copolymer. These spectra verify the successful synthesis of the HLB particles, more specifically, the copolymer of divinylbenzene and N-vinylpyrrolidone, which is a very important functional polymer for retention of broad analytes (polar as well as non-polar). The thermal stability of the developed patch is up to 384 °C; beyond that the patch starts to degrade. The initial 2.01% degradation (weight loss) is due to moisture loss from the patch's surface. The stability at high temperature confirms the integrity of the patch, which can be loaded into an inert tube and placed into the thermal desorber tray, where the analytes will be desorbed thermally. This will eliminate liquid desorption, thereby supporting the principle of green chemistry. The proposed technique is also advantageous for sample processing, where sampling and preconcentration were combined in a single step that minimizes the solvent use in comparison with the conventional solvent-based extraction process. However, the overall workflow of this technique still includes certain solvents like ACN, hexane and high energy consuming instrument like GC–MS/MS, which reduces the overall sustainability. Therefore, the solvent-free sample preparation technique is more environmentally sustainable than the whole analytical workflow, while the BAGI and AGREE scores provide an indicative assessment of analytical greenness of the method. Moreover, the present study is a proof of concept for solvent free sample preparation technique in the medical field. Future work may focus to increase the environmental sustainability for clinical sample processing.

A single concentration of McReynolds standard was used for comparative performance evaluation and analytical method optimization, rather than validation of the technique for quantitative estimation. Six analytes with different log *P* values, polar to non-polar, have been extracted by the developed patch due to its balanced hydrophobic and hydrophilic interaction efficiency (Reyes-Garcés et al. 2014). Polar compounds such as pyridine and 1-pentanol were extracted by the hydrophilic N-vinylpyrrolidone moiety, which can form hydrogen bonds and dipole–dipole interactions, whereas non-polar compounds such as benzene and moderately polar compounds such as 2-pentanone were extracted by the hydrophobic divinylbenzene unit, which is capable of forming π-π interactions (Grandy et al. 2018). This functional copolymer (HLB) has the versatility to interact with different compounds with diverse physicochemical properties compared with polyacrylate, a sorbent which extracts only hydrophilic compounds (Endo et al. 2011). The fiberglass-supported patches exhibited an effective extraction efficiency to capture the volatile and semi-volatile metabolites for bacterial metabolite profiling. However, future studies should consist of a direct quantitative comparison of the extraction efficiency of the fabricated fiberglass-coated microextraction patches and commercially available patches.

In the extraction kinetics of the TF-SPME membrane, temperature plays an important role (Mandal et al. 2024). Increasing the temperature increases the diffusion coefficient of analytes in the sample matrix, accelerating their mass transfer to the TF-SPME membrane and decreasing the equilibrium time. However, at higher temperatures, the extraction of the analytes decreases because of the exothermic nature of the sorbent (HLB) particles (Anies et al. 2025). High thermal energy weakens the intermolecular forces between the sorbent and the analytes in the sample matrix, thereby shifting the equilibrium toward desorption. Increasing the temperature enhanced the desorption rate, whereas at lower temperatures it decreased. During optimization, the developed TF-SPME patch showed the highest extraction efficiency at 25 °C, but it has the ability to extract the compounds at higher temperatures with acceptable sensitivity. The optimal temperature for bacterial growth is 37 °C, where bacteria become metabolically most active. To use this temperature for metabolites extraction is more physiologically meaningful. At 37 °C, metabolite extraction will be influenced by lowering the partition coefficient of a few polar and semi-volatile compounds between the TF-SPME coating and the aqueous phase. This results in a lower extraction efficiency of the patch in comparison to 25 °C. On the other hand, the volatility of the metabolites will increase at higher temperatures, that will enhance the recovery of the less volatile metabolites. Overall, it provided a balance between analytical performance and physiological relevance by emphasizing the bacterial metabolic profile generated under optimal temperature. The ionic strength study was not performed for analytical method optimization for this study, as it could alter the bacterial metabolite emission due to salt-in and salt-out effect. As the volatility of the metabolites is directly related to the temperature of the media, it is important to study the temperature of the headspace mode using the microextraction patch. Further experiments should be performed for analytical method optimization with both headspace and direction immersion modes.

The developed patch has been used to extract *B. cepacia-*specific metabolites during the growth phase of the bacteria. The bacterial metabolites are produced continuously during the growth phase of the bacteria and are dynamic in nature. The extended time period (8 h) was used for collecting the metabolites, as it is associated with the logarithmic growth phase of the bacteria. Future studies may consist of short-term method development for metabolic profiling to effectively identify the bacterial metabolites. Long-time exposure also increases the probability of extracting the metabolites with low volatility. In headspace, all volatile compounds were detected, whereas in direct immersion, mostly polar volatile and semi-volatile compounds were detected. In this study, ACN was used as a desorption solvent as it is compatible with GC–MS analysis and provides effective solvation capability for a wide range of volatile metabolites. The presence of heteroaromatic, ketone, and aldehyde compounds in the bacterial media may serve as an alternative pathological target for the identification of *B. cepacia* species. In this study, no single metabolite was reported as an exclusive identifier of *B. cepacia* bacteria. Rather, future studies are required for species-level identification through discriminating metabolite patterns. This metabolic profile can help with targeted analysis to identify *B. cepacia* species in clinical samples with reducing extraction time. In the present study, no internal standard was used as the work consisted of the development of a proof-of-concept to design a microextraction patch and integrate it into bacterial culture media for qualitative metabolomics profiling rather than quantitative analysis. In addition, the retention time indices through the alkane standards experiments were not calculated. The compounds were putatively determined through the NIST library after GC–MS/MS analysis, where the base peaks and major fragment ions were considered for manual interpretation. Base peaks and fragmented peaks were considered for matching with the library. As the primary motivation of this study was to develop an analytical technique for detecting *Burkholderia cepacia*, future studies will include further validation of the reported compounds using individual standards (Sumner et al. 2007).

As the study aimed to establish the development of a cost-effective microextraction patch for metabolic profiling, the analytical parameters, including LOD and LOQ, were not determined for further analytical validation with McReynolds standard. However, future studies should focus on the estimation of the accuracy of the method utilizing the fabricated patches. Although carbon mesh-supported HLB/PDMS-coated TF-SPME patches were previously reported, the novelty of this study lies in the fabrication of cost-effective and disposable TF-SPME patches for bacterial metabolite profiling. The present study is a proof-of-concept investigation of the developed patch for metabolomics, where clinical samples have not been explored. Further validation with clinical specimens will be required before applying it in clinical research. After validation, this analytical technique may facilitate future clinical research for the identification of bacterial species. Furthermore, the cost-effectiveness, along with its biodegradability, will make it more convenient for disease screening and reduce the risk of environmental pollution. In the near future, this proposed technique, coupled with a portable mass spectrometer, can help in the bedside identification of *B. cepacia*.

## Supplementary Information

Below is the link to the electronic supplementary material.

ESM 1 (DOCX 407 KB)

## Data Availability

Data will be available on request.
