# Supplementary material for: Design of solid phase microextraction patches for the detection of volatile metabolites associated with Burkholderia cepacia
Source: Appl Microbiol Biotechnol. 2026 Jul 15;110:303. doi: 10.1007/s00253-026-13962-3 (PMC13650103; doi:10.1007/s00253-026-13962-3)
Supplement: Supplementary file 1 [file 253_2026_13962_MOESM1_ESM.docx]

**Design of solid phase microextraction patches for the detection of volatile metabolites associated with *Burkholderia cepacia***

Sk Rahabar Ali^a^, Debsmita Mandal^b^, Shyam Sundar Nandi^c^, Chiranjit Ghosh^b*^, Chiranjay Mukhopadhyay^a*^

^a^Department of Microbiology, Kasturba Medical College, Manipal Academy of Higher Education, Manipal, India

^b^Manipal Institute of Technology, Manipal Academy of Higher Education, Manipal, India.

^c^ICMR-National Institute of Virology, (Mumbai unit), Indian Council of Medical Research, Haffkine Institute Compound, A. D. Marg, Parel, Mumbai 400012, India.

*** Joint corresponding authors**

A. Chiranjit Ghosh: chiranjit.ghosh@manipal.edu

B. Chiranjay Mukhopadhyay: chiranjay.m@manipal.edu

**1. Environmental Sustainability of the proposed technique:**

Table S1: Parameter for BAGI score calculation

| **SL. No.** | **Parameters** | **Methods** |
| --- | --- | --- |
| 1. | Type of analysis | Screening |
| 2. | Multi- or single-element analysis | Multielement analysis for >15 elements. |
| 3. | Analytical technique | Sophisticated instrument (GC-MS) used. |
| 4. | Simultaneous sample preparation | >95 |
| 5. | Sample preparation | Miniaturized sample preparation (SPME). |
| 6. | Samples per h | 2-4 |
| 7. | Reagents and materials | Need to be synthesized in the lab with common instrumentation and in a simple way. |
| 8. | Preconcentration | Preconcentration required, required sensitivity is met with one-step preconcentration. |
| 9. | Degree of automation | Semi-automated with common device |
| 10. | Amount of sample | <100 µL bioanalytical sample; <10 mL food/ environmental samples. |

**2. AGREEprep Report:**

Figure S1: AGREEprep Score for sample preparation.

**2.1. The 12 principles of GAC are as follows:**

- Direct analytical techniques should be applied to avoid sample treatment.
- Minimal sample size and minimal number of samples are goals.
- In situ measurements should be performed.
- Integration of analytical processes and operations saves energy and reduces the use of reagents.
- Automated and miniaturized methods should be selected.
- Derivatization should be avoided.
- Generation of a large volume of analytical waste should be avoided and proper management of analytical waste should be provided.
- Multi-analyte or multi-parameter methods are preferred versus methods using one analyte at a time.
- The use of energy should be minimized.
- Reagents obtained from renewable source should be preferred.
- Toxic reagents should be eliminated or replaced.
- The safety of the operator should be increased.

**3. Analysis of reported compounds:**

(b)

(a)

Figure S2: a. Classification of the reported compounds, b. number of similar compounds present in both direct immersion and headspace.
